# Non-viral approaches for gene therapy and therapeutic genome editing across the blood–brain barrier

**DOI:** 10.1007/s44258-023-00004-0

**Published:** 2023-07-11

**Authors:** Ruosen Xie, Yuyuan Wang, Jacobus C. Burger, Dongdong Li, Min Zhu, Shaoqin Gong

**Affiliations:** 1 Department of Ophthalmology and Visual Sciences, University of Wisconsin-Madison, Madison, WI 53705 USA; 2 Wisconsin Institute for Discovery, University of Wisconsin-Madison, Madison, WI 53715 USA; 3 Department of Biomedical Engineering, University of Wisconsin-Madison, Madison, WI 53715 USA; 4 Department of Chemistry, University of Wisconsin-Madison, Madison, WI 53706 USA

**Keywords:** Blood–brain barrier, Gene therapy, Genome editing, Non-viral vectors, Brain-targeting strategies

## Abstract

The success of brain-targeted gene therapy and therapeutic genome editing hinges on the efficient delivery of biologics bypassing the blood–brain barrier (BBB), which presents a significant challenge in the development of treatments for central nervous system disorders. This is particularly the case for nucleic acids and genome editors that are naturally excluded by the BBB and have poor chemical stability in the bloodstream and poor cellular uptake capability, thereby requiring judiciously designed nanovectors administered systemically for intracellular delivery to brain cells such as neurons. To overcome this obstacle, various strategies for bypassing the BBB have been developed in recent years to deliver biologics to the brain via intravenous administration using non-viral vectors. This review summarizes various brain targeting strategies and recent representative reports on brain-targeted non-viral delivery systems that allow gene therapy and therapeutic genome editing via intravenous administration, and highlights ongoing challenges and future perspectives for systemic delivery of biologics to the brain via non-viral vectors.

## Highlights

- Overcoming the blood-brain barrier is key to treating central nervous system disorders with gene therapy.
- Non-viral delivery systems offer a promising alternative to traditional viral vectors.
- The review covers various strategies for enhancing brain-targeted gene therapy via non-viral delivery systems and future challenges.

## Introduction

Central nervous system (CNS) disorders, including neurodegenerative diseases such as Alzheimer’s disease (AD) and Parkinson’s disease, neurodevelopmental disorders such as autism spectrum disorder and Rett syndrome, brain cancers, and stroke, impact hundreds of millions of people globally [1, 2]. As many CNS disorders are linked to genetic mutations, DNA- or RNA-based gene therapy and CRISPR-based therapeutic genome editing offer promising solutions for treating these diseases. However, the development of systemic therapies for brain disorders has been limited by the challenges of bypassing the blood–brain barrier (BBB) that regulates the homeostasis of the brain through tightly regulated neurovascular units comprising endothelial cells, pericytes, and astrocytes [3]. Therefore, the success of brain-targeted gene therapy and therapeutic genome editing relies on the effective delivery of biologics, such as nucleic acids and genome editors, across the BBB. Nucleic acids and genome editors have poor chemical stability in the bloodstream and extracellular spaces, have low cellular uptake capability, and are naturally excluded by the BBB, thereby requiring advanced vectors administered systemically for intracellular delivery to brain cells such as neurons.

The development of a safe and efficient delivery vector is a critical engineering challenge for gene therapy and therapeutic genome editing [4]. Both viral and non-viral vectors have been created so far for systemic delivery. Viral vectors, including adeno-associated viruses (AAV), adenoviruses, lentiviruses, retroviruses, and herpes simplex viruses, have been developed and used for preclinical and clinical gene therapies of CNS diseases. While the BBB poses a formidable obstacle, some recombinant AAVs, such as AAV-AS [5], Anc80L65 [6], AAV.PHP.B [7], AAV-F [8], AAV.PHP.eB [9, 10], and AAV.CPP [11], have been created for systemic delivery to achieve gene therapy and genome editing in the brain parenchyma. However, despite their high transfection efficiency, the in vivo applications of viral vectors are often hindered by several factors, including their costly manufacturing processes, limited packaging capacity, and safety concerns regarding genotoxicity and immunogenicity (Table 1). In particular, for therapeutic genome editing, the viral vector-mediated persistent expression of genome editors increases the likelihood of off-target editing effects, which may lead to cancers, and thus therapeutic genome editing requires non-viral vectors for transient expression or function of the genome editor [12].

**Table 1:** Limitations associated with viral vectors

Limitation	Explanation
Manufacturing	There is still lacking a robust manufacturing process to satisfy the need and produce affordable AAV-based therapeutics for patients [13]. For example, Luxturna, an AAV-based gene therapy designed to treat an uncommon type of hereditary blindness, was granted approval by the FDA in 2017. Despite being portrayed as a restorative therapy that brings back vision, it carries a high price tag of $850,000 [14]
Packaging capacity	AAV has a ~ 4.7 kb packing capacity. For *Sp*Cas9-based editing machinery, two separate vectors are needed to package *Sp*Cas9 and guide RNA, respectively, which impacts the genome editing efficiency [15–17]. Viral vectors with larger packaging capacity than AAV, such as lentivirus (~ 9.7 kb packing capacity), are more amenable to ex vivo treatments instead of in vivo ones due to biosafety concerns [18]
Genotoxicity	Retrovirus and lentivirus induce the integration of the transgene into the host genome and disrupt normal functional genes [12]. For example, in one study, 25% of patients treated with mouse Moloney retroviruses developed leukemia [17]. In addition, AAV-mediated persistent expression of CRISPR genome editors increases off-target editing effects [19]
Immunogenicity	Wild-type AAV results in priming of the immune system against the virus, with the development of both humoral and T cell immunity [20]. Pre-existing immunity to viral capsids also affects the safety and efficiency of viral vectors [21]. Besides, viral vector-induced persistent expression of CRISPR-Cas9 increases anti-Cas9 immune responses [22, 23]

Non-viral vectors offer a promising alternative to address the limitations of viral vectors in brain-targeted gene therapy and therapeutic genome editing through systemic administration [2, 3, 24–26]. Non-viral vectors can overcome limitations associated to viral vectors, including production difficulties, packaging size, and safety concerns. Besides, non-viral approaches enable the delivery of a variety of biologics other than DNA, such as messenger RNA (mRNA), antisense oligonucleotide (ASO), small interfering RNA (siRNA), and CRISPR-Cas ribonucleoprotein (RNP). Synthetic non-viral vectors, such as polymer- or lipid-based ones, provide versatility in engineering physicochemical and mechanical properties, including targeting ligand, ligand density, internal or external stimuli-responsiveness, size, surface charges, shape, and stiffness (Table 2) [2]. These properties play crucial roles in the transport of vectors from the bloodstream, across the BBB, to the brain [2].

**Table 2:** Properties of non-viral vectors that affect their transport to the brain

Property	Mechanism and example
Targeting ligand	See details in Sect. 3 in this review
Ligand density	Ligand density substantially affects the brain accumulation of non-viral vectors. For example, Kataoka et al. reported a synthetic micelle system conjugated with different densities of glucose (0, 10, 25, and 50%, mol%) with 25% exhibiting the highest brain accumulation of the micelle [27]
Stimuli-responsiveness	Internal stimuli such as pH (endosomal pH at 5.5–6.5) [28, 29] and GSH (cytosolic GSH concentration at 1–10 mM) [30–33] have been used to facilitate nanoparticle disassembly/degradation in the brain cells and thus the release of payloads. External stimuli such as ultrasound [34–36] have been used to temporarily disrupt the BBB and/or enhance the brain accumulation of the non-viral vectors
Size	Previous studies found densely PEGylated nanoparticles with diameters ≤ 100 nm can exhibit faster spreading and larger diffusion volume in the mouse brain parenchyma in vivo after intracranial injections, compared to their counterparts with larger diameters. However, even so, the diffusion distance of the nanoparticles was still very limited in the brain parenchyma, typically around 1 mm surrounding the injection site. [37, 38]
Surface Charges	After intravenous administration, cationic nanoparticles are rapidly cleared with the shortest half-life, followed by anionic nanoparticles, whereas neutral and slightly negative charged nanoparticles have the longest half-lives in circulation [39]. Moreover, it is reported that PEGylated or negatively charged surface can help nanoparticle diffusion in the brain [37, 40, 41]
Shape	Most non-viral vectors are spherical, but shape affects the interaction of nanoparticles with cells [2]. Mitragotri et al. reported rod-shaped particles exhibited higher BBB transport than sphere-shaped ones in an in vitro BBB model [42]

*GSH* Glutathione, *PEG* Polyethylene glycol

In this review, we aim to provide a comprehensive overview of recent advances in the development of non-viral delivery systems for gene therapy and therapeutic genome editing targeting the brain. The importance of this review lies in the fact that effective delivery of biologics to the brain remains a significant challenge due to the presence of the BBB. We start with a brief review on the structure of the BBB. Then, we discuss various brain targeting strategies and representative non-viral brain-targeted delivery systems for gene therapy and therapeutic genome editing via intravenous administration (Table 3). Finally, we highlight the important challenges and opportunities in the development of non-viral vectors for safe and efficient systemic delivery of biologics to the brain.

**Table 3:** Representative publications discussed in this review

Strategy	Ligands	Materials	Size (nm)	Payload biologics	Animal model(s)	Ref
Tf receptor	CPP + Tf	Liposome	150	pDNA	Healthy wild-type mice	[43, 44]
Tf receptor	CPP + Tf	Liposome	160	pDNA	AD mouse model	[45]
nAChR	RVG	Peptide	N/A	siRNA	Healthy wild-type mice, EGFP reporter mice, and encephalitis mouse model	[46]
nAChR	CPP + RVG	Silicon-silicate	180	siRNA	Brain injury mouse model	[47]
nAChR	RVG	Peptide	100	siRNA	Brain injury mouse model	[48]
nAChR	RVG	Polyplex	150	pDNA	Healthy wild-type mice	[49]
nAChR	RVG	Polyplex	110	pDNA	AD mouse model	[50]
nAChR	RVG	SPION/polymer hybrid	63	Cas9/sgRNA pDNA	AD mouse model	[51]
LRP1	Angiopep-2	Polyplex	167	pDNA	GBM mouse model	[52]
LRP1	Angiopep-2	Polymeric nanocapsule	25	siRNA	GBM mouse model	[53]
LRP1	Angiopep-2	Polymeric nanocapsule	31	Cas9/sgRNA RNP	GBM mouse model	[31]
LRP1	Angiopep-2	Polyplex	149	Cas9/sgRNA RNP	GBM mouse model	[54]
GLUT1	Glucose	Polyplex	45	ASO	Healthy wild-type mice	[55]
GLUT1	Galactose	Polyplex	118	siRNA	AD mouse model	[56]
GLUT1	Glucose	Liposome	179	pDNA	Healthy wild-type mice	[57, 58]
GLUT1	Glucose	Silica nanocapsule	51	pDNA, mRNA, Cas9/sgRNA RNP	Healthy wild-type or Ai14 reporter mice	[32]
CPP	LIMK2 NoLS peptide	Polyplex	91	pDNA	Glioma mouse model	[59]
CPP	mHph3 + iRGD	Polymeric nanogel	95	Cas9 protein + minicircle DNA	Glioma mouse model	[29]
Neurotransmitter	Neurotransmitter	Lipid nanoparticle	100	ASO	Healthy wild-type or Ai14 reporter mice	[60]
FUS	None	Microbubble	3,600	pDNA	Healthy wild-type mice	[61]
FUS	Folate	Microbubble	2,300	pDNA	GBM rat model	[62]
FUS	None	Polyplex	56	pDNA	Healthy wild-type rats	[63]
FUS	None	Polyplex	50	pDNA	Parkinson’s disease rat model	[64]
FUS	None	Polyplex	50	pDNA	GBM mouse model	[65]
FUS	None	Lipid nanoparticle	93	mRNA	Healthy wild-type mice	[66]
Exosome	RVG	Exosome	88	siRNA	Healthy wild-type mice	[67]

*Tf* Transferrin, *nAChR* Nicotinic acetylcholine receptor, *CPP* Cell-penetrating peptide, *LRP1* Low-density lipoprotein receptor-related protein 1, *GLUT1* Glucose transporter 1, *GBM* Glioblastoma, *pDNA* Plasmid DNA, *mRNA* Messenger RNA, *siRNA* Small interfering RNA, *sgRNA* Single guide RNA, *RNP* Ribonucleoprotein, *ASO* Antisense oligonucleotide, *FUS* Focused ultrasound

### BBB

**Fig. 1:**
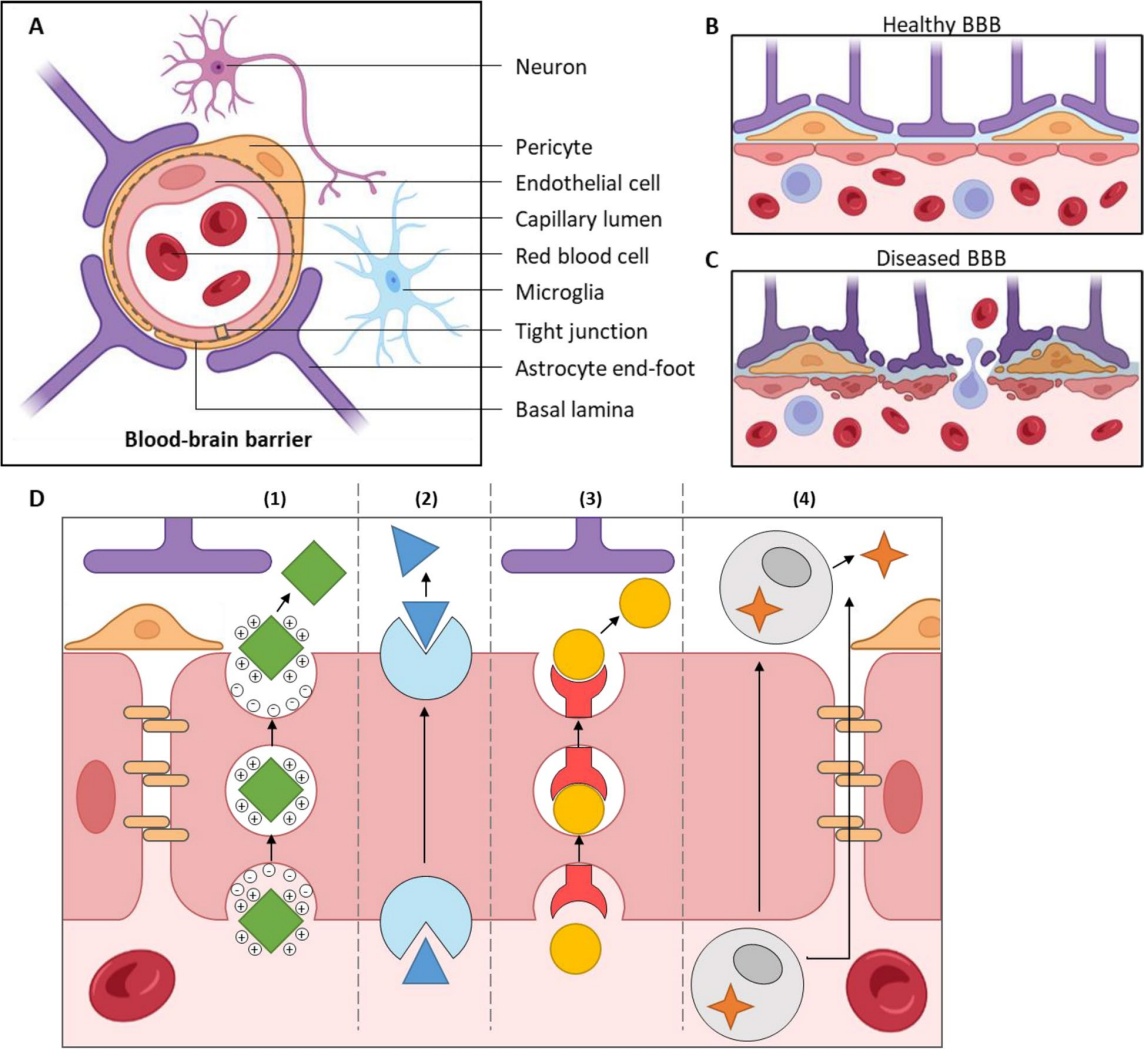
Structure of the BBB. (**A**) The human brain has a dense network of blood vessels with large-diameter arterioles and a microvasculature called the neurovascular unit, composed of a single layer of tightly connected endothelial cells. The endothelial cells are covered by basal lamina, pericytes, and astrocyte end-foot processes, and are innervated by neuronal synapses and microglial cells. The structural components and cellular interactions of the neurovascular unit are crucial for the development and maintenance of the BBB. (**B**) In a healthy brain, intact BBB can limit the passive transport of substances into the brain parenchyma. (**C**) In a diseased brain, the functions of BBB can be compromised, and vascular permeability is thus increased. More monocytes and macrophages can infiltrate the brain parenchyma than in the normal state. (**D**) Several pathways for molecules to cross the BBB which can be used for brain delivery, including (1) adsorptive-mediated transcytosis, (2) carrier-mediated transcytosis, (3) receptor-mediated transcytosis, and (4) cell migration

The BBB is formed by the tight junctions between the specialized endothelial cells lining the blood vessels of the brain (Fig. 1) [2, 26, 68, 69]. The tight junctions between the endothelial cells in the BBB create a continuous and sealed barrier that restricts the movement of substances between the bloodstream and the CNS. Additionally, the BBB's endothelial cells are surrounded by pericytes, which provide mechanical stability and regulate blood flow, and astrocyte end-feet, which help regulate the chemical environment of the CNS and maintain the structural integrity of the BBB. These different cell types and tight junctions create a highly selective barrier that allows certain molecules to pass while restricting others. Certain molecules, such as oxygen, carbon dioxide, and lipophilic molecules smaller than 400 Da can diffuse passively across the barrier. Carbohydrates, amino acids, and hormones are transported across the BBB with the help of corresponding transporters on the endothelial cells, while macromolecules such as insulin and transferrin rely on receptor-mediated transport. Endothelial ion transporters and channels also play a crucial role in controlling ion concentrations in the CNS. Lastly, efflux mechanisms using ATP-binding cassette transporters can actively pump drugs and drug conjugates, xenobiotics, and endogenous metabolites from the CNS to the blood, contributing to the barrier function. However, there are certain conditions, such as brain injury or CNS disease, that can cause the BBB to become more permeable, leading to an increased exchange of substances between the blood and the CNS, such as glioma, AD, Parkinson’s disease, and stroke (Fig. 1b, c).

There are several ways for molecules to cross the BBB which can be used for brain delivery (Fig. 1d) [2, 26]. (1) In adsorptive-mediated transcytosis, positively charged proteins, peptides or molecules interact with negative glycocalyx and cell membranes on the BBB cells' luminal side, and trigger endocytosis to form transcytotic vesicles. These vesicles move to the abluminal membrane of the BBB cells, fuse with the membrane, and release the molecules into the brain. (2) In carrier-mediated transcytosis, the therapeutic molecules bind to a carrier (e.g., amino acid transport system) and are transported across the BBB [70]. (3) In receptor-mediated transcytosis, the therapeutic molecules bind to a specific receptor (e.g., insulin receptor, transferrin receptor) on the luminal side of the endothelial cells, triggering endocytosis to form transcytotic vesicles that are trafficked to the brain. This method is the most extensively studied route for brain delivery [71]. (4) Cell migration, such as through monocytes or macrophages, can also cross the BBB, either through transcytosis or the pericellular space, and release proteins or viroid particles into the brain.

### Strategies for bypassing the BBB

Existing strategies for bypassing the BBB to deliver therapeutic agents to the CNS include targeting specific receptors and transporters, as well as using physical and biological mechanisms. In this section, we will discuss these strategies: (1) receptor-mediated transcytosis using ligands targeting transferrin (Tf) receptor, nicotinic acetylcholine receptor (nAChR), low-density lipoprotein receptor-related protein 1 (LRP1), glucose transporter 1 (GLUT1), and neurotransmitter receptors; (2) non-receptor-mediated transport facilitated by cell-penetrating peptide; (3) physical strategies using focused ultrasound; and (4) biological strategies like exosomes. Each of these strategies has unique characteristics that make them promising for delivering therapeutics to the CNS.

### Tf receptor

One strategy to overcome the BBB for nanoparticle delivery to the brain involves targeting the membrane-bound Tf receptor on brain capillary endothelial cells (BCECs) [72, 73]. The Tf receptor functions to internalize transferrin-bound iron from the blood when intracellular iron levels are low. Tf receptor is highly expressed on the cell surface of BCECs and internalizes transferrin-bound iron via receptor-mediated endocytosis. Targeting this receptor is predominantly done by functionalizing nanoparticle surfaces with the 80 kDa Tf protein. Many studies have demonstrated Tf-conjugated nanosystems can overcome the BBB using Tf conjugation and subsequent Tf receptor-mediated transcytosis for drug delivery to the brain [74, 75].

Singh et al*.* developed Tf-modified nanosystems to deliver nucleic acid therapeutics to the brain [43–45]. Here, a dual-ligand functionalized liposomal carrier was first developed to deliver pDNA across the BBB [43]. The liposomal nanocarrier conjugated with a CPP penatratin (Pen) and Tf exhibited an average size of ~ 150 nm and zeta potential around + 20 mV. The dual-ligand functionalized liposomes were tested in an in vitro BBB model, which was constructed by bEnd.3 (brain endothelial cells) and rat primary astrocytes and showed significantly higher efficiencies in bypassing the BBB and transfecting rat primary neurons afterwards (~ 7% GFP-positive neurons), compared to liposomes conjugated with only Tf or CPP (both with ~ 3% GFP-positive neurons). These in vitro results were supported by further in vivo studie*s*, where the intravenously injected dual-ligand functionalized liposomes delivered GFP and β-galactosidase pDNA to the brain of healthy wild-type mice with significantly higher transfection efficiencies than un-conjugated or single ligand-functionalized liposomes. Singh et al*.* later expanded upon their earlier work and tested the therapeutic potential and effectiveness of these brain-targeting CPP-Tf-liposomes in a mouse model of AD [45]. Here, they investigated the delivery of a nerve growth factor gene (NGF). Following intravenous administration of NGF pDNA-loaded CPP-Tf-liposomes, the treated mice displayed significantly lower soluble and insoluble amyloid-β (Aβ) deposits in brain tissue, compared with mice treated with naked NGF pDNA and untreated mice. In another study, Singh et al*.* further investigated the effect of different types of CPPs conjugated onto liposomal carriers together with Tf on the transfection efficiency in the brain across the BBB. Three types of CPPs were studied, namely, a vascular endothelial-cadherin-derived peptide, a pentapeptide QLPVM, and an HIV-1 trans-activating protein (TAT) peptide [44]. Liposomes functionalized with TAT peptide and Tf exhibited ~ twofold higher transfection efficiencies in bEnd.3 endothelial cells, glial cells and primary neurons in vitro, compared to liposomes functionalized with other ligands studied. In vivo biodistribution study in healthy wild-type mice indicated that ~ 7.7% injected dose per gram (ID/g) brain accumulation 24 h after intravenous injection of the fluorescently labeled and dual-ligand functionalized liposome. The authors attributed this finding to several factors: (1) The high arginine content in the TAT peptide enabled more efficient endosomal escape of the payload, possibly due to endosomal membrane disruption by the positively charged residues. (2) The TAT sequence improved the nanoparticles’ interactions with the cell membrane and subsequently increased the interaction of Tf with the Tf receptor due to the closer proximity of the ligand and receptor. This study further demonstrates the benefits of dual-ligand functionalization for brain targeted delivery.

### nAChR

The nAChR is highly expressed and widely distributed in the central nervous system [46]. A 29-amino-acid peptide derived from rabies virus glycoprotein (RVG) (termed RVG peptide) can bind nAChRs on both BCECs and neurons [46]. RVG peptide can induce receptor-mediated transcytosis to penetrate through the BBB and finally shuttle the biologics to the brain parenchyma. RVG peptide has been successfully used as the brain targeting ligand to deliver small molecule drugs, siRNA, and proteins for diagnosis and/or therapeutics (reviewed elsewhere [76, 77]) for various diseases, such as GBM, traumatic brain injury, and ADs.

Manjunath et al*.* engineered an RVG peptide for brain targeted delivery of siRNA in 2007 [46]. The RVG peptide was modified by adding nonamer arginine residues at its carboxy terminus, forming the RVG-9R peptide. siRNA complexed with RVG-9R peptide was able to bind with almost 100% of Neuro2a cells, but less than 10% of other cell types studied (e.g., HEK293T, HeLa, etc.). After intravenous injection into mice, siRNA/RVG-9R peptide complexes were delivered to the neuronal cells in the cortex, striatum, and thalamus, resulting in specific gene silencing within the brain. The RVG-9R-mediated delivery of siRNA targeting superoxide dismutase type 1 (SOD1) led to ~ 50% reduction of SOD1 production in the mouse brain. Furthermore, intravenous treatment with RVG-9R-bound antiviral siRNA afforded robust protection against fatal viral encephalitis in mice. Repeated administration of RVG-9R-bound siRNA did not induce inflammatory cytokines or anti-peptide antibodies. Mice treated with (1) rabies virus matrix (not RVG) peptide + antiviral siRNA, or (2) RVG-9R + control siRNA, or (3) in the control group (no treatment) all died within 10 days, while mice treated with RVG-9R + antiviral siRNA resulted in ~ 80% survival rate after 30 days. Thus, RVG-9R peptide provides a safe and noninvasive approach for the delivery of siRNA and potentially other therapeutic molecules across the BBB.

The RVG peptide has been widely used for the delivery of small molecule drugs, peptides and proteins, and nucleic acids to the brain [77]. For instance, Sailor et al. reported a biodegradable and intrinsically photoluminescent calcium silicate-coated porous silicon nanoparticle for siRNA delivery with a high siRNA loading content (~ 20 wt%) [47]. The combination of RVG peptide and myristoylated transportan (a type of CPP) in these nanoparticles was able to effectively target cells and achieve gene knockdown in vitro. In Neuro2a cells, the dual-ligand conjugated nanoparticles showed improved siRNA delivery and thus gene knockdown efficiency (~ 51%) compared to particles with only RVG (~ 36%). A similar result was found in vivo in a brain injury mouse model after intravenous injection of these nanoparticles, as qualitatively studied by IVIS. Bhatia et al. also demonstrated that RVG conjugated with transportan can complex with siRNA and form nanocomplexes (60–100 nm in diameters) [48]. Intravenously injected nanocomplexes in a mouse model of brain injury showed ∼80% knockdown of Caspase 3, which is a therapeutic target known to contribute to apoptosis after traumatic brain injury, in the damaged region of the injured hemisphere.

RVG peptides have also been utilized for pDNA delivery to the brain. For example, Jiang et al. reported an RVG peptide-modified and PEGylated polyamidoamine (PAMAM) dendrimers that formed 150-nm-sized nanoparticles by complexing with pDNA [49]. Mice intravenously injected with the nanoparticles loaded with luciferase pDNA showed higher expression of luciferase (744 U/mg protein) in the brain compared to unmodified nanoparticles (431 U/mg protein) at 48 h post-administration. In 2016, the same research group reported another nanocarrier for AD treatment. PEGylated dendrigraft poly-L-lysines was utilized to deliver pDNA (encoding antisense transcript to downregulate β-secretase 1 (BACE1)) and D-peptide (D-TLKIVW, all-D amino acid inhibitor to disrupt the p-tau associated fibril formation) to the brain through intravenous injection [50]. Similar to the PAMAM dendrimer, the dendritic amine-rich structure of dendrigraft poly-L-lysines provides abundant reaction sites for peptide conjugation and positive charges to complex pDNA. Successful co-delivery of pDNA and peptide overcoming the BBB by RVG peptide was demonstrated both in vitro and in vivo. Approximately 30% downregulation of BACE1 transcript was detected in the hippocampi of the APP/PS1 transgenic mice after four injections of the nanoparticle, and thus led to the decrease of amyloid deposits in the cortex and hippocampus. The tau-positive signals in the brain were also ~ 60% lower in the mice treated with nanoparticles conjugated with D-peptide than saline-treated mice. The nanoparticle also caused behavior improvement and restoration of cognitive function in the treated mice as studied in a Morris water maze test.

Zhang et al*.* reported that RVG peptides can facilitate CRISPR-Cas9 delivery to the brain in 2021 [51]. They constructed traceable nano-biohybrid complexes (F-TBIO) to efficiently deliver CRISPR-Cas9 genome editor and a small molecule drug (fluvastatin) simultaneously into brain lesions for treating AD and enhancing MRI simultaneously. The nanosystem, F-TBIO, was constructed with a superparamagnetic iron oxide nanoparticle (SPION) core, which is conjugated with polylysine-PEG with RVG or fluvastatin terminals. F-TBIO can be complex with CRISPR-Cas9/sgRNA pDNA via electrostatic interactions, resulting in a nanocomplex with 63 nm in hydrodynamic diameter*. *In vivo studies in an AD mouse model revealed that ten or three intravenous injections of Cas9/sgRNA pDNA-loaded nanosystem targeting the *Bace1* gene downregulated ~ 20% of the expression of BACE1 in the mice and reduced ~ 50% of the amyloid-β plaque area in the hippocampus of 2xTg-AD mice. As a result, the cognitive abilities of the treated 2xTg-AD mice were improved, as studied by Y maze and Morris water maze experiments.

### LRP1

As one of the identified receptors that allow receptor-mediated transcytosis, LRP1 is a single-pass transmembrane receptor with a large size (600 kDa) that is highly expressed in the CNS, including brain endothelial cells and GBM cells [78]. Angiopep-2, a 19-amino-acid peptide, was developed based on the sequence alignment of aprotinin and other LRP1-binding proteins with a Kunitz-type domain [78]. Angiopep-2 can cross the BBB, and thus has been employed to deliver biologics to the brain, particularly in the treatment of GBM.

In 2016, Gao et al. introduced a polymer called PPA, which is composed of poly (L-lysine)-grafted polyethyleneimine (PEI-PLL) combining high transfection efficiency of PEI and the good biodegradability of PLL [52]. The backbone of PPA was further conjugated with PEG and Angiopep-2. The optimal formulation of PPA for pDNA delivery was determined and characterized, with a 5:1 weight ratio of PPA/DNA complex yielding ~ 167 nm in hydrodynamic diameter and + 20 mV in zeta-potential. The transfection efficiency, cytotoxicity, and cellular uptake mechanism of the PPA/DNA nanoparticle were studied in U87MG cells in vitro. In vivo imaging studies demonstrated that PPA/DNA nanoparticles could penetrate the BBB and accumulate in the striatum and cortex in the brain via systemic administration, although no quantitative analysis was reported. Moreover, pDNA encoding Herpes simplex virus type I thymidine kinase, delivered using the PPA/DNA nanoparticle, inhibited tumor cell proliferation and induced apoptosis. The effectiveness of PPA/DNA treatment in treating glioma was confirmed by a noticeable reduction in tumor size (by over 2/3) 21 days after treatment and an extended median survival time (from 26 days with PBS injection to 37 days with PPA/DNA treatment) in an invasive orthotopic human GBM mouse model.

Utilizing a similar targeting strategy, Shi’s and Zheng’s groups collaborated and published a series of studies reporting non-viral delivery approaches for siRNA or CRISPR-Cas9 for GBM treatment through systemic administration [31, 53, 54]. They developed a safe and efficient RNAi agent, an Angiopep-2-functionalized intracellular-environment-responsive siRNA nanocapsule to boost siRNA-based GBM therapy [53]. The nanocapsule was prepared by an in-situ free radical polymerization using siRNA as a template, positively charged acrylate guanidine as monomers, and GSH-cleavable N,N′-bis(acryloyl) cystamine as crosslinkers, and acrylate PEG. The resulting small (~ 25 nm) siRNA polymeric nanocapsule was then conjugated with Angiopep-2 for BBB crossing and GBM targeting. The siRNA nanocapsule had a longer plasma elimination half-life than naked siRNA (46 min vs. 5 min) in the bloodstream. Facilitated by Angiopep-2, nanocapsules accumulated 6.69% ID/g in the tumor, a 2.7-fold increase compared to non-ligand ones, in an orthotopic U87MG xenograft mouse model. The Angiopep-2-conjugated siRNA nanocapsule also led to enhanced median survival time of the GBM-bearing micro from 26 days (treated with non-ligand counterparts) to 42 days, without adverse effects.

**Fig. 2:**
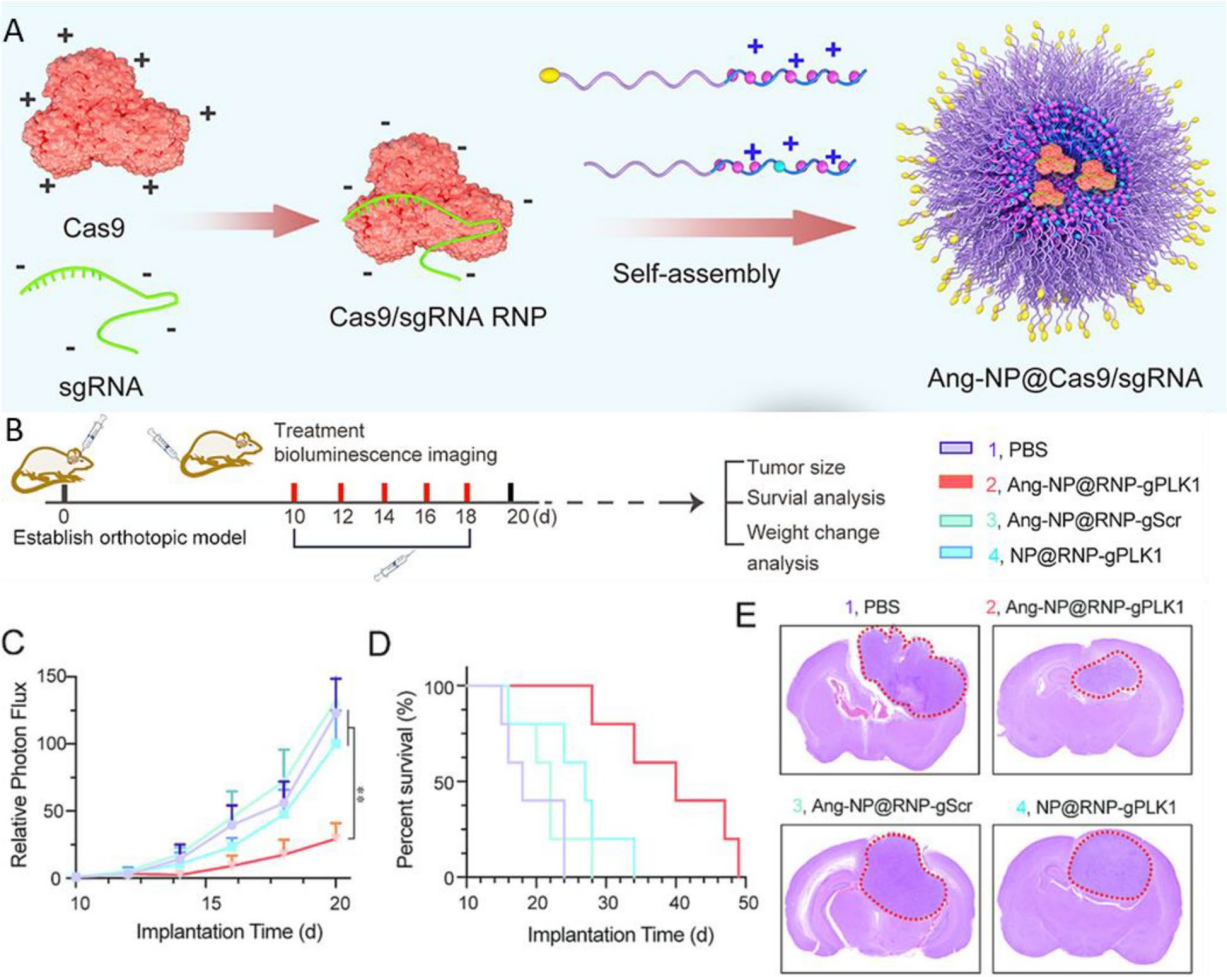
**A** Schematic illustration of the Cas9/gRNA RNP polymeric nanoparticle delivery system and its application to in vivo glioblastoma therapy. **B-E** In vivo treatment with Ang-NP@RNP-gPLK1 and relevant controls in the U87MG-Luc orthotopic mouse glioma model. **B** Experimental timeline for the administration of nanocomplexes and various assessments. **C** Quantified luminescence levels in mice using the Lumina IVIS III system showing tumor volumes. **D** Mouse survival curves (n = 5). **E** H&E staining of whole brain excised on day 22. Figures were reproduced from [54] with permission from Elsevier

A more recent study described the creation of a nanocapsule for efficient encapsulation of CRISPR-Cas9 for noninvasive GBM targeting via systemic administration [31]. The CRISPR-Cas9 nanocapsules were fabricated by encapsulating the Cas9/sgRNA RNP within a GSH-degradable polymer shell using in situ polymerization, a similar process as previously reported [79]. The Angiopep-2 peptide was included as the targeting ligand, and consequently, the Cas9 RNP nanocapsule showed effective GBM tissue targeting (11.8% ID/g) and efficient *Plk1* gene editing in the tumor (28.6% indel by Sanger sequencing) in a GBM mouse model. This treatment also extended the median survival time of the tumor-bearing mice from 24 to 68 days. Another study reported an Angiopep-2 peptide decorated and guanidinium and fluorine functionalized polymethacrylate-based nanoparticle for Cas9 RNP delivery for the treatment of GBM (Fig. 2) [54]. The inclusion of guanidinium and fluorine functional groups in the polymer structure facilitated its interaction with Cas9 RNP and maintained the nanoparticle stability in the bloodstream without impairing Cas9 activity. In addition, by leveraging the Angiopep-2 peptide, the nanoparticles efficiently crossed the BBB and accumulated in brain tumors (12.9% ID/g). The Cas9-mediated knockout of the *Plk1* gene in the tumor in an orthotopic GBM mouse model effectively suppressed tumor growth, significantly improved the median survival time of mice from 18 days (PBS-treated) to 40 days, and meanwhile had negligible side effects.

### GLUT1

Kataoka et al. first reported a novel strategy to bypass the BBB by leveraging the glucose transporter-1 (GLUT1)-mediated transcytosis through glycemic control [27]. GLUT1 is abundant on the cell membrane surface of the BCECs and has an essential role in the glucose supply to the brain [80]. By controlling the blood glycemic levels through 16–24 h of fasting, the density of GLUT1 on the luminal side of the BCEC membrane increases. When the blood glucose is rapidly restored, GLUT1 on the luminal side promptly translocates to the abluminal side of the BCEC membrane, presumably through a transcytosis process [27, 55]. This unique mechanism facilitates the transcytosis of systemically injected glucose-modified nanoparticles, allowing them to bypass the BBB and reach the brain parenchyma under the condition of glycemic control. The binding and dissociation balance of glucose-modified nanoparticles with GLUT1 on BCECs is crucial in controlling the transcytosis process. The low affinity of glucose to GLUT1 is advantageous because the density of glucose ligands on the nanoparticle surface can be adjusted to control binding/dissociation balance.

Based on their findings, Kataoka et al. engineered a glucose-modified polymeric nanocarrier for the noninvasive delivery of antisense oligonucleotide (ASO) to the brain via intravenous injections [55]. The optimized nanocarrier, having a size of around 45 nm and a desirable glucose density (50% molar blending ratio of glucose-conjugated polymer and regular polymer, leading to ~ 52 glucose ligands per micelle), efficiently accumulated in the whole brain (~ 7% ID/g) one hour after intravenous administration. A single intravenous injection led to significant knockdown of a target long non-coding RNA in various regions of the brain, such as the cerebral cortex (~ 54%), hippocampus (~ 20%), and thalamus (~ 37%). These results showcase the potential of glucose-modified polymeric nanocarriers for the noninvasive administration of ASOs in the treatment of CNS disorders.

Shi et al. reported a glycosylated “triple-interaction” polymeric siRNA nanomedicine that can effectively downregulate BACE1 expression for the treatment of AD [56]. The nanoparticle, which has a size of 118 nm and a polymer/siRNA weight ratio of 2.5:1, is composed of a diblock copolymer formed by PEG and polymethacrylate with guanidinium and fluorine side groups, similar to the one designed for Cas9 RNP delivery [54]. Instead of glucose, galactose was conjugated to the nanoparticle surface to allow efficient penetration of the BBB via glycemia-controlled GLUT1–mediated transport so that the encapsulated siRNA can be delivered to the brain to decrease BACE1 expression and reduce amyloid plaque levels. The conjugation of galactose led to 5.8-fold increase in the nanoparticle accumulation in the brain than the non-galactose modified one after intravenous injections. In an APP/PS1 transgenic AD mouse model, 10 times of intravenous injection of the nanoparticles decreased ~ 20% of the BACE1 expression, leading to reduced levels of Aβ plaques and improved cognitive capacity in AD mice without any side effects.

Singh et al. developed a liposomal nanocarrier for the delivery of pDNA to the brain [57, 58]. The surface of these liposomes was modified with mannose (a GLUT-1 substrate), CPP (i.e., penetratin), and/or RVG peptide to enhance brain targeted delivery. Compared to unmodified liposomes, the ligand-modified liposomes exhibited significantly higher pDNA transfection efficiency in primary astrocytes and primary neurons and more effective transport across the BBB in vitro. Both studies used C57BL/6J wild-type mice and showed in vivo pDNA delivery to the brain. A single intravenous administration resulted in over 50% increase in expression level of the protein encoded by the pDNA compared with untreated mice, with no signs of inflammation or toxicity. However, these studies did not use glycemic control which may enhance the delivery efficiency.

**Fig. 3:**
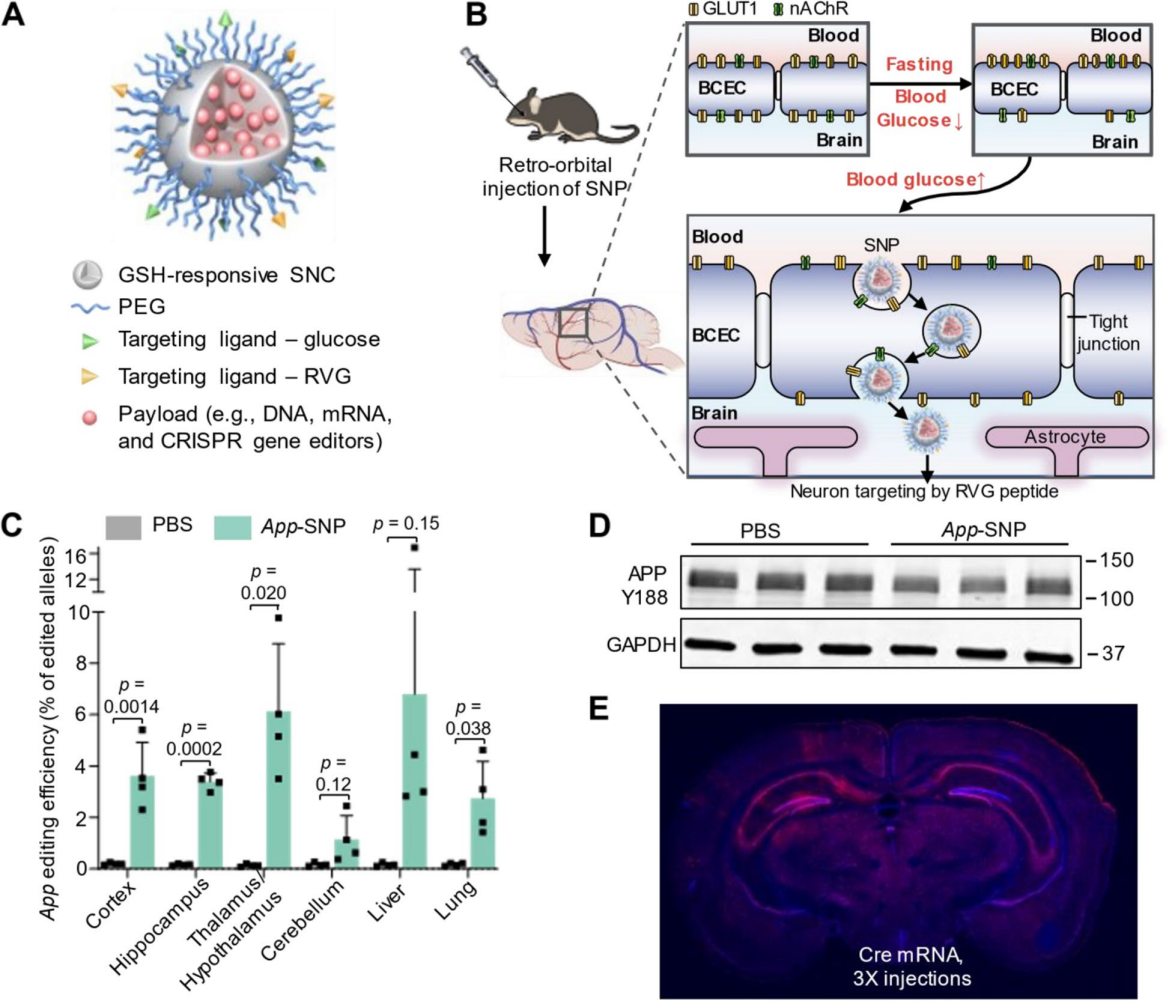
Overcoming the BBB for gene therapy via systemic administration of GSH-responsive silica nanocapsules. **A** Schematic illustration of GSH-responsive SNC for brain delivery of various biologics via systemic administration. **B** Schematic illustration of systemic delivery of SNCs into the brain via the dual targeting ligand strategy. **C** *App *editing efficiency in different brain regions and major organs by deep sequencing after intravenous injection of *App*-SNC loaded with CRISPR-Cas9 mRNA+sgRNA targeting App. **D** Brains of *App*-SNC-treated mice were immunostained by APP Y188 antibody recognizing the APP C-terminus and analyzed by western blot. **E** Cre mRNA-encapsulated brain-targeting SNC achieved brain-wide transfection/editing. Representative coronal section mosaic tile confocal microscope images of the brains of Ai14 mice with three intravenous injections of SNC was shown. Blue, DAPI staining nuclei. Red, tdTomato. Figures were reprinted and adapted from [32] with permission from WILEY.

We recently developed a library of GSH-responsive silica nanocapsules (SNCs) and screened them for brain targeting via systemic administration (Fig. 3) [30, 32]. The top-performing SNC had a hydrodynamic diameter of 51 nm and a net neutral surface charge. In vivo studies demonstrated that SNCs conjugated with 10% glucose and 10% RVG peptide (targeting nAChR and GLUT-1, respectively) under glycemic control efficiently bypassed the intact BBB after intravenous injections. This allowed for brain-wide delivery of various biologics, including mRNA, Cas9 mRNA/sgRNA, and Cas9/sgRNA RNP, and brain-wide genome editing targeting both reporter genes (i.e., Ai14 stop cassette) and disease-relevant endogenous genes (i.e., amyloid precursor protein (*App*) and tyrosine hydroxylase (*Th*) genes) in Ai14 reporter mice and wild-type mice, respectively. In particular, we achieved up to 28% neuron editing via systemic delivery of Cre mRNA in Ai14 mice, and up to 6.1% editing of the *App* gene (resulting in 19.1% reduction in the expression level of intact APP) in wild-type mice via triple-injection. Besides, the excellent biocompatibility of SNCs was systematically evaluated and demonstrated in vivo by various assays, including blood biochemical parameters, inflammatory cytokine levels, and histological studies. The SNCs have a high degree of versatility and modularity, which allows them to accommodate and deliver various CRISPR genome editors, such as base editors and prime editors. The ease of targeting different genes using the CRISPR system further enhances the potential applications of the SNCs for treating a broad range of neurological disorders.

### CPP

CPPs and CPP-conjugated nanovectors, possess unique capabilities to facilitate the translocation of membrane-impermeable molecules, such as siRNA, mRNA, and pDNA, into cells by crossing cell membranes [81, 82]. Recent research also suggested that CPP-conjugated nanovectors could mediate the delivery of therapeutic agents across the BBB [83, 84]. Huang et al. constructed a nanosystem comprising of dendrigraft PLL, PEG, and a CPP derived from the nucleolar translocation signal sequence of the LIM Kinase 2 protein, for the delivery of pDNA across the BBB [59]. The conjugation of CPP enhanced the cellular uptake and achieved higher transport efficiency across BBB in vitro. The nanosystem was further evaluated in an orthotopic glioma-bearing mouse model, where the pDNA encoding the inhibitor of growth 4 was delivered for glioma suppression. The CPP-conjugated nanosystem effectively induced apoptosis in the tumor and extended the median survival time from 29 days (saline treated) to 47 days of glioma-bearing mice.

Zhou et al. designed liposome-templated hydrogel nanoparticles (LHNPs) to deliver the CRISPR-Cas9 genome editor to the brain [29]. The LHNP, decorated with iRGD and mHph3 CPPs to enhance the intracellular delivery efficiency, resulted in 2.6 times more accumulation in the brain tumor compared to the LHNP without iRGD modification. To enhance brain accumulation, Lexiscan, a drug that can temporarily increase BBB permeability, was encapsulated in the LHNP. The inclusion of Lexiscan increased nanoparticle accumulation in the brain tumor 2.1-fold. Additionally, LHNP-mediated delivery of minicircle DNA encoding sgRNA targeting *Plk1* gene decreased the cell viability from 47.2% to 20.7% in U87 cells, and from 41.7% to 19.8% in GS5 cells, compared with LHNP-mediated delivery of pDNA encoding sgRNA targeting *Plk1* gene. The LHNP treatment led to a 60.4% decrease in PLK1 expression in the tumor site and significantly inhibited tumor growth and prolonged the median survival time from 29 days (saline treated) to 40 days in tumor-burdened mice.

### Neurotransmitter

Xu et al. reported the integration of neurotransmitter (NT)-derived lipidoids in solid lipid nanoparticles (LNPs) enhanced brain delivery through intravenous injection [60]. NTs are endogenous chemicals that facilitate neurotransmission and some of them can cross BBB through active transport. The lipidoids, which contain moieties of NT derivatives, were synthesized by Michael addition reaction between the primary amine of the NTs and acrylate-containing hydrophobic tails. With five injections of NT-lipidoid-doped LNPs loaded with ASO (1 mg/kg) against tau proteins, ~ 50% reduction of tau mRNA and ~ 30% reduction of tau protein expression were observed. Moreover, GFP-Cre fusion protein delivered by NT-lipidoids-doped LNP led to tdTomato expression in the cortex, hippocampus, and cerebellum in the treated Ai14 mice. However, future studies are needed to further evaluate the potential of NT-lipidoids for the delivery of pDNA, mRNA, and genome editors to the brain for gene therapy applications.

### FUS

**Fig. 4:**
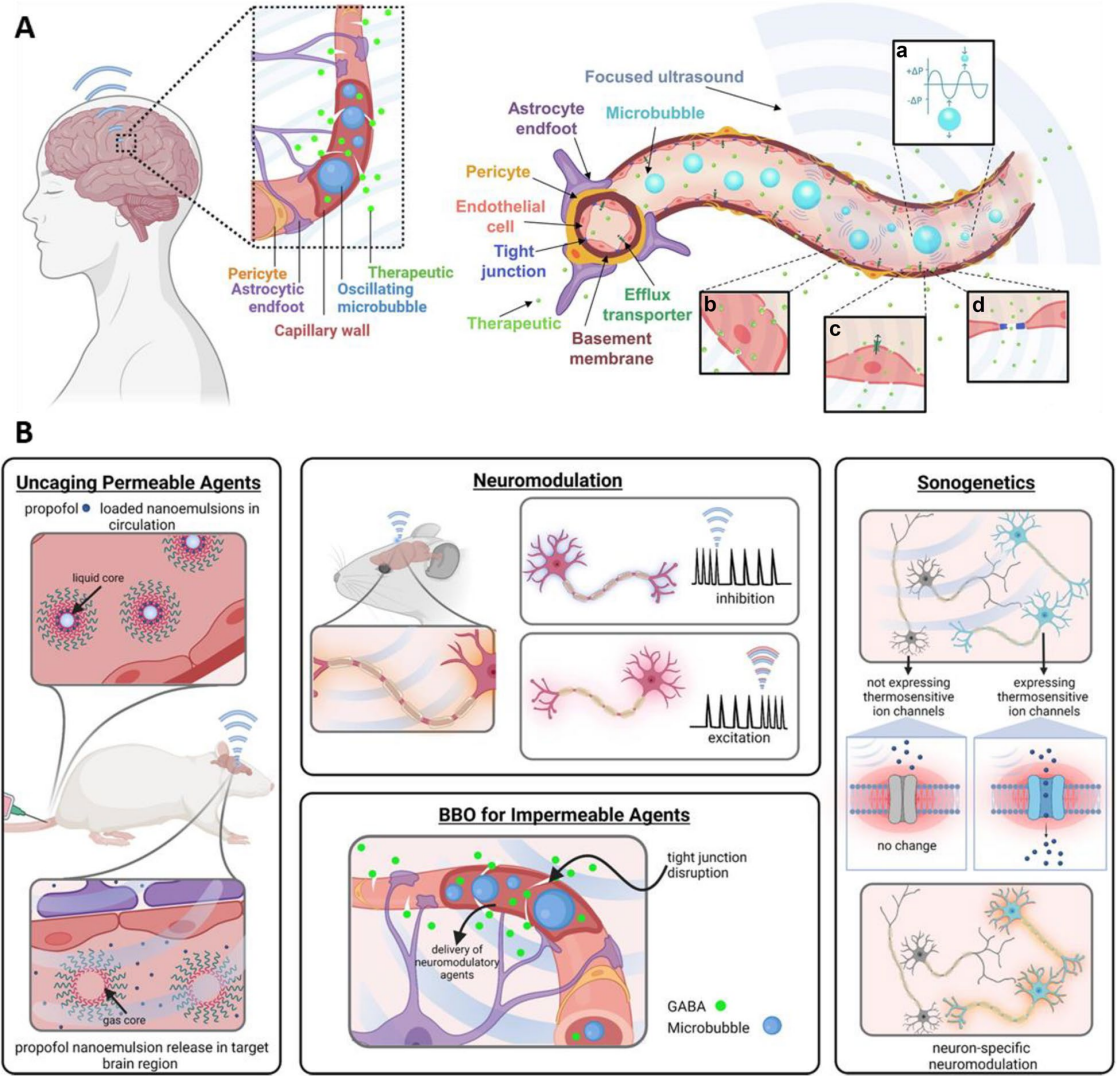
**A** The physiology of the BBB, and the permeabilization of the BBB with microbubbles and FUS. **a** FUS induced volumetric oscillations of microbubbles. **b** Transcytosis of a therapeutic through an endothelial cell. **c** Sonoporation of an endothelial cell. **d** Disruption of tight junctions between adjacent endothelial cells. **B** A summary of the neuroscience tools being studied at the pre-clinical level which utilize FUS for various neurological applications. Figures were reproduced from [35] with permission from Elsevier

Microbubble-assisted FUS has gained attention as a promising technique for delivering therapeutic agents to the brain (Fig. 4). By intravenously administering microbubbles and then irradiating the target location of the brain with FUS, oscillation or cavitation energy is produced in the blood vessels of the irradiated site, inducing transient BBB opening and allowing systemically administered therapeutics to get into the brain [85]. FUS has been used for delivering a variety of diagnostic or therapeutic agents, including magnetic resonance imaging (MRI) contrast agents, small molecule drugs, and nucleic acids, as reviewed in previous publications [34, 35, 85].

Early efforts focused on the use of cationic microbubbles for nucleic acid delivery to the brain facilitated by FUS [61, 63, 85, 86]. These cationic microbubbles can bind to anionic nucleic acid through electrostatic interactions. Because nucleic acids are adsorbed on the surface of microbubbles, nucleic acids can be immediately exposed to the vasculature being disrupted by FUS-activated microbubbles, resulting in nucleic acid extravasation and trans-BBB delivery. For instance, Cheng et al*.* developed a DNA-loaded microbubble for brain delivery via intravenous injection [61]. With the aid of MRI-guided FUS, transient BBB disruption and internalization of pDNA into the neurons were observed. At 48 h after sonication, the expression of GFP was observed in some neurons, and the GFP expression level in the FUS-treated region was enhanced tenfold that of the control group without microbubbles. In another study, Yeh et al*.* reported a folate-conjugated pDNA-loaded cationic microbubble for brain delivery [62]. With FUS, these microbubbles can be converted into vesicles with nanometer size (~ 140 nm in diameter) that help pDNA transport into the brain parenchyma.

The major limitations of cationic microbubbles include limited delivery efficiency, the need for high doses of pDNA, and stability issues with the microbubble-pDNA complex in the bloodstream [35, 85, 87]. Recent advancements in delivery methods take advantage of the combination of microbubbles and nanoparticles to transport therapeutic agents to the brain. By utilizing microbubbles to temporarily open the BBB under FUS, nanoparticles carrying biologics payloads can cross the disrupted BBB and deliver payloads to the targeted brain site. Price et al*.* developed a series of strategies using brain-penetrating nanoparticles (BPNs) combined with MRI-guided FUS and microbubbles for gene therapy in the brain through intravenous injection [63–65]. The BPNs, approximately 60 nm in diameter, were formulated with a cationic polymer, PEI, coated with dense PEG to prevent nanoparticle adhesion to extracellular matrix components in the brain. In healthy wild-type rats, systemically administered BPNs delivered pDNA through the BBB and led to a dose-dependent transgene expression in the FUS-treated region [63]. The transgene expression was observable after 24 h post-administration and lasted for over 28 days. Over 42% of all cells, including neurons and astrocytes, were transfected with pDNA in the FUS-treated region, compared to less than 6% of brain cells transfected in the contralateral non-FUS-treated hemisphere. This method showed no signs of toxicity or astrocyte activation. In a follow-up study, BPNs loaded with glial cell-line derived neurotrophic factor (GDNF) pDNA were used to treat Parkinson’s disease in a 6-OHDA-induced rat model [64]. After a single injection of BPNs, widespread and uniform GDNF expression throughout the targeted brain region lasted over 10 weeks. The treatment restored both dopamine levels and dopaminergic neuron density and reversed behavioral indicators of disease-associated motor dysfunction with no signs of toxicity. BPNs were also developed for gene delivery to treat glioma due to their ability to penetrate both the BBB and blood-tumor barrier [65]. It was found that the FUS-mediated opening of the BBB and blood-tumor barrier doubled the mean interstitial flow velocity magnitude and thus increased the dispersion of BPNs (volume transfected by pDNA) through brain tumor tissue by over 100%.

The strategy of combining FUS/microbubbles and nanoparticles was extended to mRNA delivery in the brain using LNP. In a recent study, Kawakami et al*.* studied FUS/microbubble-assisted BBB opening for the intravenous delivery of mRNA-loaded LNP to the brain [66]. The optimal intensity of FUS irradiation was determined to be 1.5 kW/cm^2^, which efficiently opened the BBB without causing harm such as hemorrhage or edema. The mRNA expression, as measured by luciferase, was only observed in the FUS-irradiated side of the brain and the luciferase expression occurred only when FUS and microbubbles were applied. The mRNA-mediated protein expression was faster but shorter-lived compared to pDNA. However, mRNA-mediated protein expression was only observed in the microglia and endothelial cells, but not in astrocytes or neurons.

### Exosomes

Exosomes are another emerging and promising nanotechnology for new therapeutics and diagnostics. These cell-derived, small vesicles with a diameter of 40–100 nm are actively secreted by most cell types [26]. Their biocompatibility, stability in the bloodstream, natural origin and surface composition, ability to evade phagocytosis and the immune system, and most importantly, their innate ability to traverse the BBB, make exosomes ideal candidates as non-viral vectors for gene delivery to the brain. Moreover, exosomes can interact with target cells through receptor interaction or direct membrane interaction, enabling effective delivery of the payloads. In 2011, Wood et al*.* developed dendritic cell-derived exosomes that can deliver siRNA to the brain in mice [67]. The brain-targeting capability of exosomes was achieved by engineering the RVG peptide on the exosome surface. siRNA was then loaded into the extracted and purified exosomes through electroporation. Upon intravenous injection, the RVG-modified exosomes delivered GAPDH siRNA specifically to neurons, microglia, and oligodendrocytes in the brain, leading to specific gene knockdown. The therapeutic potential of exosome-mediated siRNA delivery was demonstrated by the strong mRNA (60%) and protein (62%) knockdown of BACE1, a therapeutic target of AD in wild-type mice. So far, while many exosomes have been developed for siRNA and miRNA delivery to the brain, as recently reviewed [88, 89], there have been few reports of pDNA or mRNA delivery to the brain using exosomes, likely due to the difficulty of encapsulating large nucleic acids into exosomes. The potential of exosomes for delivering pDNA, mRNA, and genome editors for treating CNS diseases is yet to be demonstrated.

### Existing challenges and outlook

There is a need to further enhance the efficiency and specificity of non-viral vectors for brain targeted delivery. It is known that non-viral vectors often have a lower transfection/genome editing efficiency compared to viral vectors such as AAV [10, 11], because AAV can exploit the natural infection cycle of the virus to enter the cell nucleus and integrate into the host genome [90, 91]. Additionally, the specificity of the delivery using current non-viral vectors has a lot of room to improve. Accumulation and delivery of biologics to off-target organs, such as the liver, spleen, and lung, is a common and almost inevitable issue associated with intravenously injected nanoparticles [92]. Furthermore, nanoparticles that accumulate in the brain tend to transfect/edit many types of brain cells including neurons, astrocytes, endothelial cells, and other cell types. This will likely not be a concern when therapeutic genome editing aims to disrupt inherited gene mutations, as these genetic mutations occur throughout the whole body. For certain types of neurodegenerative diseases, gene therapy or genome editing in peripheral organs in addition to the brain may even be beneficial. To achieve organ- and/or cell-specific gene therapy and genome editing, pDNA with cell-specific promoters (such as neuron-specific promoters) or other emerging new cell specific genome editors can be applied. To improve the delivery efficiency and specificity in the brain, novel strategies for bypassing the BBB and transport within the brain should be explored. The materials' chemistry/formulation should also be further studied through high-throughput screening and optimization methods.

It is important to highlight that most published work on in vivo experiments for brain delivery has been conducted in mouse and rat models. There are several major differences between rodents and humans that can impact the effectiveness, safety, and scalability of delivery methods. These differences include (1) differences in brain anatomy, including the BBB, which can impact the effectiveness of the strategies to bypass the BBB and transport vectors within the brain parenchyma [93–95]; (2) differences in metabolic processes and immune responses, which can affect the safety and efficacy of the delivery of therapeutics agents [96, 97]; and (3) differences in the brain volumes between species – a relatively large nanoparticle diffusion area in the mouse brain may be negligible in the human brain when considering the respective brain volumes [94]. Besides these major differences between rodents and humans, it should be noted that current animal disease models cannot fully and faithfully reproduce human diseases, despite the use of advanced genetic engineering technology for producing relatively sophisticated animal models [98]. Therefore, future in vivo studies should focus on more complex and/or humanized mouse models, or non-human primate models, for preclinical evaluations of brain delivery systems.

Due to the BBB, delivering therapeutic biologics to the CNS is challenging both technically and clinically. There are only a limited number of delivery systems that have progressed to human clinical trials (Table 4). This limited progress can be partially attributed to the significant differences in size and complexity between the human body and the rodent models employed for testing various drug delivery systems. Despite the appealing potential of non-viral delivery systems for treating CNS diseases [99, 100], and recent advancements in this field, the majority of the developed non-viral vectors for genome editing in the brain have relied on invasive administration methods, such as intracranial or intracerebral ventricular injections [33, 101–106]. These methods have shown promise in preclinical studies, but they are not practical for widespread clinical use and carry the risk of significant side effects, especially with repeated treatments for certain diseases. In addition, the genome-edited area in the brain is typically restricted to the area near the injection site through these local and invasive routes. The development of safe and effective non-viral vectors for gene therapy and therapeutic genome editing in the brain through non-invasive and systemic administration routes remains a critical area of ongoing research.

**Table 4:** Examples of non-viral systems for the CNS delivery in clinical trials

Drug name	Material	Disease	Delivery route	Trial register	Status
MTX110	Panobinostat nanoparticle	Glioma	Convection-enhanced delivery	NCT04264143	Recruiting
APH-1105	Nanoparticle	AD	Intranasal	NCT03806478	Not yet recruiting
DepoCyte	Liposome	Brain metastases	Intrathecal	NCT00854867	Completed
Cytarabine	Liposome	Brain metastases	Intrathecal	NCT00992602	Completed
Doxorubicin	Liposome	Brain tumor	Intrathecal	NCT00019630	Completed
Doxorubicin	PEG-liposome	GBM	Intrathecal	NCT00944801	Completed
AGuIX	Polysiloxane nanoparticle	Brain metastases	Intravenous	NCT02820454, NCT03818386, NCT04094077, NCT04899908	Completed Recruiting Terminated Recruiting
EnGeneIC EDV	Nanocell	GBM/Astrocytoma	Intravenous	NCT02766699	Recruiting
RNA-LP	RNA-loaded DOTAP liposome	GBM	Intravenous	NCT04573140	Recruiting
Gliadel	Polymer wafer	Metastatic brain cancer	Parenchymal	NCT00525590	Completed
CNM-Au8	Gold nanoparticle	Amyotrophic lateral sclerosis	Oral	NCT04098406	Completed
CNM-Au8	Gold nanoparticle	PD	Oral	NCT03815916	Completed
NU-0129	Spherical nucleic acid gold nanoparticle	GBM/Gliosarcoma	Intravenous	NCT03020017	Completed
Ferumoxytol	SPION	Brain neoplasms	Intravenous	NCT00769093	Terminated
ARCT-810	LNP	Ornithine transcarbamylase deficiency	Intravenous	NCT05526066	Recruiting

## Data Availability

Not applicable.
